# Does langenskiold staging have a good prognostic value in late onset tibia vara?

**DOI:** 10.1186/1749-799X-7-23

**Published:** 2012-06-07

**Authors:** Ashraf Ahmad Khanfour

**Affiliations:** 1 Damanhour National Medical Institute, Ali El-Garim St. In front of Omar Afandi stores, Bohera state, Rasheed, Egypt

**Keywords:** Tibia vara, Langenskiold, Ilizarov, Prognosis

## Abstract

**Background:**

Although many literature studied the effect of many factors on the prognosis of the early-onset Blount disease, studies that were written on the prognostic factors affecting late onset tibia vara are still limited.

**Purpose:**

The aim of this study is to evaluate the prognostic value of the Langenskiold classification system for late onset tibia vara.

**Methods:**

Twenty children from the Sporting Health Insurance Student Reference Hospital – Alexandria, with a diagnosis of late onset tibia vara were evaluated for the effect of the Langenskiold staging system on the prognosis after they all had been treated by gradual correction by Ilizarov technique using the so called “juxta-articular hinge assembly” after a mean follow-up period of 4.9 years (range : 4–6, SD 0.75).

**Results:**

The difference in varus recurrence rates between the “low grade group” and “high grade group” was found to be statistically significant (p = 0.008) as will be discussed later. There was no statistically significant relation between the age of the patients at the time of operation, sex, length of the follow up period and the degree of pre-operative varus deformity angle (DA) and the recurrence (p > 0.05).

**Conclusion:**

We concluded that Langenskiold staging system is a reliable, reproducible and a good prognostic factor for late onset tibia vara.

## Introduction

Late onset tibia vara is a growth disturbance of the medial part of the proximal tibia occurring after 4 years old. The physis is involved primarily. Shortly thereafter there will be an affection of each of the epiphyseal cartilage, the secondary ossification center, the slope of the articular cartilage, and the adjacent metaphysis. Bowing of the lower extremity is the result of this disease. The primary deformity is tibial deformation characterized by a varus angulation at the proximal metaphyseal level, often with internal tibial torsion and procurvatum of the upper tibia. Thereafter, secondary deformities of the distal femur and distal leg with limb length inequality will occur [1-5].

**Figure 1:**
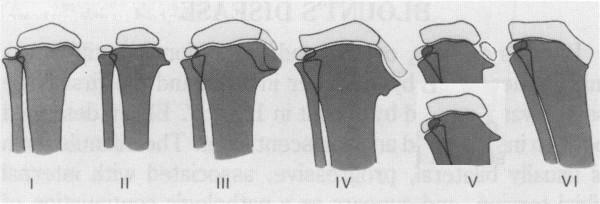
**Langenskiold stages:*Stage I*: medio-distal beaking of the upper proximal tibial metaphysis.*Stage II***: wedging of the medial part of the upper tibial epiphyseal secondary ossification center plus a saucer shaped defect of the upper surface of the metaphyseal beak due to its dissolution, fragmentation & collapse. ***Stage III***: stepping of the infero-medial border of the secondary ossification center but without extending distal to the physeal plate level plus deeping of the metaphyseal saucer into a step in the medial metaphysis. ***Stage IV***: the epiphyseal secondary ossification center passes more distally and cross distal to the physeal level to fill the metaphyseal step. ***Stage V***: separation of the most medial part of the ossification center from the bulk of the secondary ossification center and resides now in the depth of the metaphysiseal step below the physis. This is radiologically expressed as either a horizontal cleft (double epiphysis) or complete absence of the medial secondary ossification center as it will be overshadowed by the upper medial tibial metaphysis. ***Stage VI***: medial epiphyseal plate closure with a bony bridge.

In 1952, Langenskiold [1] had described the widely known 6 progressive roentgenographically visible stages that where originally described for the infantile type of Blount disease and was widely used as factor for determining its prognosis. This classification is based on a cascade of different degrees of epiphyseal depression and metaphyseal fragmentation of the proximal medial tibial epiphysis. These stages of tibia vara map out the degree of the pathological affection of the proximal tibial physio-metaphyseal region (Figure 1). This classification has been confirmed by the following numerous roentgenographic studies done on tibia vara between the years 1952 and 1963 [2,3,5,6]. 

Many literatures divided these stages into *low grade group* “stages I-IV” and *high grade group* “stages V and VI”. Although there is interobserver and intraobserver variability, it still the system most commonly used by orthopaedic surgeons till the present time [1,3,7].

Many studies in the literature demonstrated the effect of different factors on the prognosis of the early-onset Blount disease. They found a high prevalence of varus recurrence following tibial osteotomy in cases of high grade group of the Langenskiold stage, older age at the time of the osteotomy, and a lack of postoperative valgus overcorrection while the degree of the pre-operative deformity did not affect the prognosis [3,4,8,9].

Until recently, Langenskiold staging classification has not studied as a factor in determining the prognosis of late onset tibia vara [4].

The aim of this study is to evaluate the prognostic value of Langenskiold classification system for late onset tibia vara.

## Patients and methods

**Figure 2:**
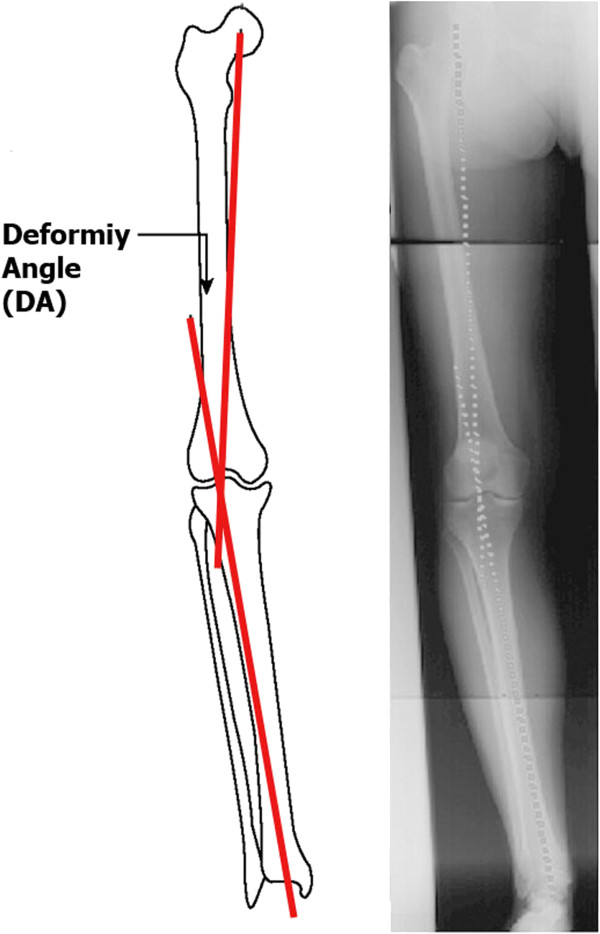
Deformity angle (DA) measurement.

Ethical permission for this study was obtained from the General Organization of Teaching Hospitals and Institutes Research Ethical Committee and informed consent was obtained from all patients and their guardians before participation in the study. This is a retrospective study including 20 children (Table 1) from the Sporting Health Insurance Student Reference Hospital – Alexandria, with a diagnosis of late onset tibia vara (older than 4 years) of age when the bowing was first noted by the family. These children were recalled for reevaluation after a minimum follow-up period of 4 years post-operatively. Two were bilateral. All cases were treated by the author in the period between December 2003 and January 2007. There were 9 males and 11 females. The two bilateral cases were males. Mean age of the patients at time of operation was 11 years (range : 8–14, SD 1.8). Ten cases were left sided, eight right sided and two bilateral. Fourteen legs were operated upon for the first time (63.6%) and eight cases were recurrences after prior treatment (36.4%). All patients that attended the follow up in this study were subjected to both clinical and roentegenograohic examination. Clinically the internal tibial torsion was measured clinically using the thigh-foot axis test. If it is present, the foot axis points inward and the angle is negative. The angle was measured with a goniometer. Roentgenographically, a long leg anteroposterior x-rays in standing position from the hip to the ankles were taken. On this x-ray both the deformity angle (DA) and limb length discrepancy were measured. The DA (Figure 2) is the angle formed between two lines, the first one from the center of the hip joint to the center of the knee joint (mechanical axis of the femur) and the second line from the center of the knee joint to the center of the ankle joint (mechanical axis of the tibia). A negative DA represents a varus deformity. This DA value was then compared with the pre-operative one. Loss of correction is the difference between the angle achieved at the end of the initial treatment and the DA done at the re-evaluation for this study. The minimal follow up was at least 4 years post-operatively. According to the Langenskiöld classification, we divided the patients into two groups: grade I, II, III, and IV as the “*low grade group*” and grade V and VI as the “*high grade group*”. The first group included 16 cases and had a mean pre-operative varus deformity of 34.7° (range: 25°-45°). The second group included 6 cases with a mean pre-operative varus deformity of 34.2° (range: 20°-45°). The preoperative internal rotation of the tibia was 10.9° (range : 0.0°-35°; SD 12.2°). The mean preoperative deformity angle was −34.5° (range : -20° to −45°; SD 7.2°). The mean pre- operative limb length discrepancy was 1.5 cm (range : 0.0-5; SD 1.5). The mean follow-up period was 4.9 years (range : 4–6, SD 0.75).

**Table 1:** Data of the patients

	**Age (Y)**	**Sex**	**Side**	**Presentation**	**Langenskiold Classification**	**Pre-Op DA**	**Pre-Op Intortion**	**Pre-Op LLD (cm)**	**Degrees of correction**	**Follow up period (Y)**	**Loss of correction**
**1**	12	male	Rt.	Recent	II	-30	-	-	40	5	20
**2**	9	Male	Bil	recent	VI	Lt. -25	-	-	35	6	35
					VI	Rt. -20			30	5	25
**3**	9	Female	Lt	Recurrent	II	-35	10	5	45	6	0
**4**	14	male	Bil-	recent	II	Rt. -40	35	-	50	6	10
					II	Lt. -25	30	-	45	5	0
**5**	11	Male	Lt	Recent	I	-40	15	2	50	4	0
**6**	12	Female	Rt.	Recent	VI	-45	-	2	55	4	10
**7**	10	Male	Rt.	Recurrent	IV	-30	-	1	40	5	15
**8**	9	Female	Lt	Recurrent	VI	-45	25	4	55	4	15
**9**	12	Female	Lt	Recent	II	-35	-	3	45	5	0
**10**	10	Female	Lt	Recurrent	VI	-40	20	2	50	5	20
**11**	12	Female	Rt.	Recurrent	IV	-40	10	1	50	6	0
**12**	10	male	Lt	Recent	III	-30	25	4	40	5	10
**13**	8	Female	Rt.	Recurrent	I	-35	20	-	45	5	10
**14**	11	Male	Lt	Recent	I	-40	25	1	50	4	15
**15**	12	Female	Rt.	Recent	III	-25	-	-	35	5	0
**16**	9	Male	Lt	Recent	VI	-30	15	-	40	5	15
**17**	13	Female	Rt.	Recurrent	I	-45	-	2	55	4	20
**18**	10	Female	Rt.	Recent	III	-40	20	3	50	6	10
**19**	14	Female	Lt	Recurrent	II	-30	-	2	40	4	0
**20**	12	Male	Lt	Recent	I	-35	-	1	45	4	20

**Figure 3:**
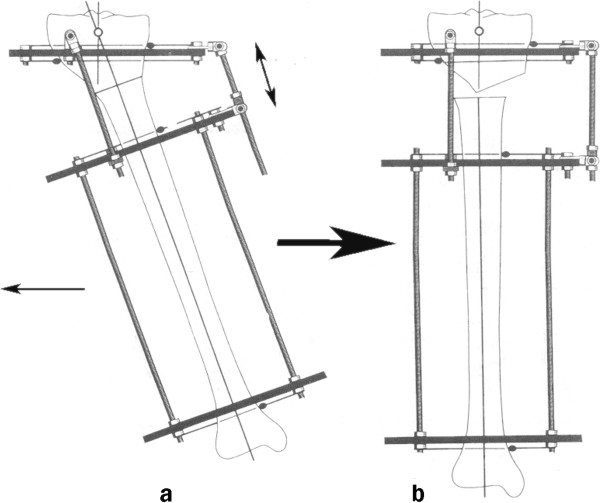
Juxta-articular hinge assembly.

Surgical technique:

Gradual correction by distraction osteogenesis using the Ilizarov technique was the standard protocol of treatment applied for all the cases included in the study. Intraoperatively, excision of part of the fibula, mounting the pre-constructed “Ilizarov juxta-articular hinge assembly (Figure 3)” on the affected tibia, single high tibial osteotomy, and acute intra-operative correction of the internal tibial torsion component of the deformity if present were done. Post-operatively, Gradual correction of the varus deformity was started one week post-operatively and took 3–5 weeks depending on the degree of the pre-operative deformity. Varus correction was accompanied with a synchronous lateral translation of the distal tibial segment on the proximal one. This translation occurs as a result of the intrinsic dynamic behavior of the construct that guide gradual correction. This happens as a result of the position of the hinge of the apparatus which lies at the level of the knee joint line proximal to the level of the tibial osteotomy. Gradual correction was continued until 10° valgus overcorrection was attained. Shortening, if present, was also corrected gradually. At the end, the frame was locked and a further 10–12 weeks were allowed for consolidation, guided by regular radiological follow-up before frame removal. If a lengthening was performed, a further 4–6 weeks were needed for every additional centimeter. The degree of surgical correction (the sum of the initial degrees of the varus deformity plus 10° valgus overcorrection) was noted after gradual correction was obtained.

In data analysis, Student’s *t*-test, Spearman’ Rank Order correlation test, and Linear regression model were used. P < 0.05 is considered significant using SPSS version 17 On IBM compatible laptop.

## Results

**Figure 4:**
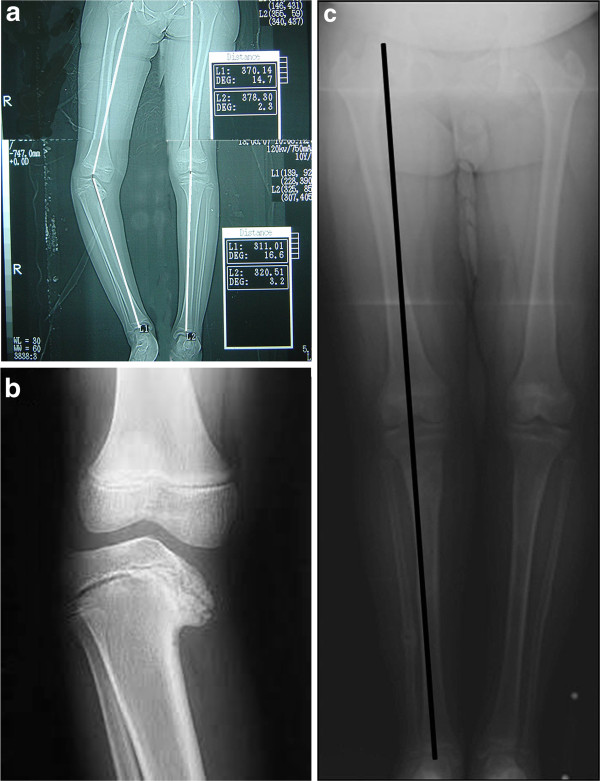
**(a) Preoperative A-P radiogram of a 12-year-old female with right sided late onset tibia vara with a preoperative DA angle of −45°. (b)** Roentgenogram of the right knee shows stage IV of the Langenskiold staging. **(c)** follow-up A-P radiogram after 4 years shows no recurrence.

**Figure 5:**
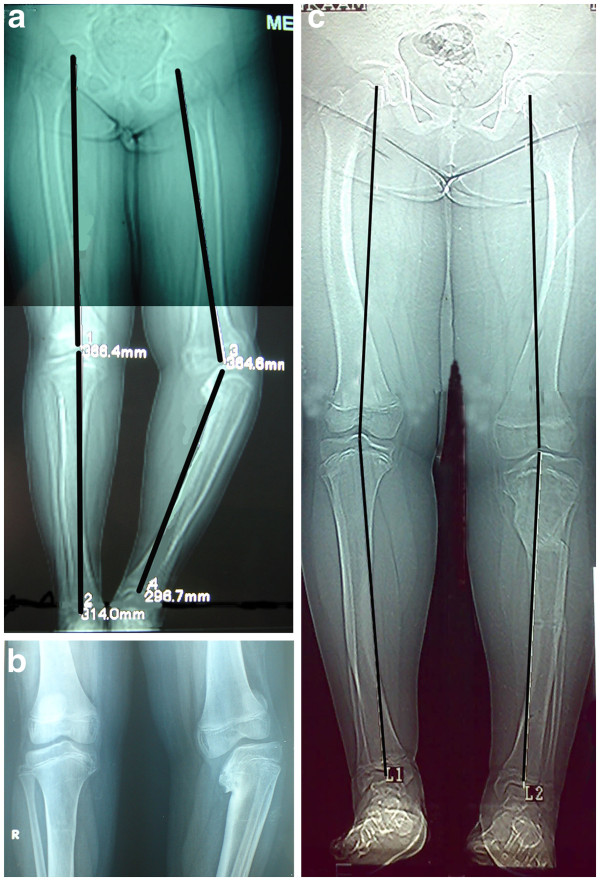
**(a) Preoperative A-P radiogram of a 9 year old male with left recurrent late onset tibia vara with a preoperative DA angle of −45°.** He had 25° of tibial internal tosion which was corrected acutely intraoperatively. **(b)** Roentgenogram of both knees shows left knee stage VI of the Langenskiold staging. (c) follow-up A-P radiogram after 4 years shows a recurrence of 15°.

The overall recurrence of varus deformity (loss of more than 10^o^ of the correction) was noted in 10 of 22 cases (45.5%) The mean angular correction was 45° (range : 30°- 55°; SD 6.9°). After a mean follow-up period of 4.9 years (range : 4–6 years; SD 0.75) (Figure 4, 5). The mean loss of correction was 11.36° (range : 0.0° - 35°; SD 9.78°). According to the Langenskiold staging, the mean varus recurrence in the “*low grade group*” was 18° (range 15°-20°, SD 2.7°) and occurred in 5 out of 16 cases (31%). While it was 22° (range 15°-35°, SD 4.8°) and occurred in 5 out of 6 cases (83%) in the “*high grade group*”. The difference in varus recurrence rates between the two groups was found to be statistically significant (p = 0.008). There was no statistically significant relation between the age of the patients at the time of operation, sex, length of the follow up period and the degree of varus deformity angle DA and the recurrence (p > 0.05). No leg length discrepancy or torsional deformities were recorded at the end of follow-up.

A Spearman's Rank Order correlation was run to determine the relationship between age, sex, pre-operative deformity angle, amount of correction, and amount of lengthening on one hand and the amount of recurrence on the other hand. There were absence to low correlation between the recurrence and each of these variables. But there was a moderate degree of positive correlation between the Langenskiold grades and the amount of recurrence. This was statistically significant (r = .521, P = .013).

## Discussion

Understanding of the natural history of tibia vara is important for its management [2,3,10-12]. According to Langenskiold, the most common complication encountered in treatment of tibia vara is recurrence [3,8]. Although most of the recent studies on late onset tibia vara concentrated on discussing different new treatment modalities for this condition, they only documented its early results [13-17] and little has been written on different factors affecting the prognosis for this challenging disease on a late follow up basis [18-20]. In reviewing the literature on late onset tibia vara, it has been found that there was a great variation in the overall recurrence rate recorded by different authors where Chotigavanichaya et al. [19] recorded a recurrence rate in 48 cases to be 83% after 6 years. Non rigid fixation may explain this high recurrence rate as in more than 2/3 of their cases, the osteotomy was fixed by crossed wires and the remainders by external fixator. Alekberov et al. [18] recorded a recurrence rate in 69 cases to be 8.7% after 6.7 years where Eidelman et al. [20] recorded no recurrence in his work on 8 cases after 3.7 years. The low recurrence rate of these last 2 series may be explained on the bases of that their cases were relatively of older ages at the time of treatment where the mean age of the cases of the former was 10.7 years and 14.6 years for the later, moreover, both authors fixed the osteotomy rigidly by circular fixators. In this work, the overall recurrence rate was 10 out of 22 cases (45.5%) which lies in the middle of the above two extremes. In order to determine the effect of the different factors on the prognosis, each of the age, sex, deformity angle and Langenskiold staging are tabulated and statistically studied. Both of the operative technique and the angle of final correction were standardized in this work for all cases where the first was “gradual angulation translation high tibial osteotomy using Ilizarov technique” and the later was 10^o^ valgoid overcorrection. While most of studies on the early form of this disease demonstrated the significance of the age as a prognostic factor with a poorer prognosis and high recurrence if osteotomy was done after 4 years [1,3,4], this was not documented in most literature on the late onset cases except by Chotigavanichaya et al. [19] that postulated a statistically significant effect of age on the prognosis of their similar cases. So, the prognosis of the infantile form of Blount disease must be considered separately from that in the late onset form [4,5,21]. Instead of the absence of consensus on the optimal degree of final correction, many literature proved the significant statistical effect of the degree of the final post-operative correction on the results where it was found to be 5^o^ -10 ^o^ valgoid overcorrection [4,18-20,22]. This factor was standardized in this work to be 10^o^ for all patients.

Although nowadays there is a growing interest for measuring the lower limbs deformities using mechanical axes deviation and joint orientations, [23-25] in this study the deformity angle (DA) method [19] was used. The advantage of using DA method instead of the radiological frontal mechanical axis analysis for Blount’ disease lies in the fact that the upper tibial epiphysis in these cases is deformed with depression of its medial plateau, which makes measuring of the knee joint orientation angles such as upper medial tibial angle less reproducible. Adding to this, the degree of the deformity may be over-estimate due to the large cartilaginous component of the upper tibial epiphysis at this age. On the other hand, while advanced imaging techniques provide more detailed information than do plain radiographs, the additional cost, radiation exposure, and potential need for sedation and general anesthesia associated with some of these modalities should be considered [3,4,18].

The results of this study demonstrated that the Langenskiold staging classification was the only factor that significantly affects the prognosis of late onset tibia vara after a mean follow up 4.9 years. Although there is interobserver and intraobserver variability of this staging system, fortunately, this variability is between the low grade stages only [7]. So, on considering the first 4 stages as a group and the last 2 as another group, eliminates this variability and make this classification system more reproducible. It was documented that the behavior of cases with low grade group I-IV “reversible epiphyseal changes” is different from high grade group V and VI “irreversible epiphyseal changes”. This was built on the observation that histological and roentgenographical studies on stages I to IV showed a typical sequel of partial and often temporary blockage that takes place in the ossification in the proximal end of the tibia in cases having tibia vara deformity with increasing age and not a sign of progression while in late cases, stage V-VI were proved to result from permanent epiphyseal-metaphyseal bony bridge with inhibition of the medial portion of the tibial growth plate [1-4]. The reversibility of stages I-IV had been proved by many authors depending on their observation that many legs with tibia vara in stages I when treated by early upper tibial osteotomy they passed into stages II, III, and IV and reached maturity without recurrence of the deformity. Also it was proved by histopathologic finding and by appearance of the corresponding roentgenographic it mimics the phenomenon of healed osteomyelitis or bone tuberculosis [3].

We can conclude that Langenskiold staging system is a reliable and a good prognostic factor for late onset tibia vara.

Depending on the above results, it is recommend considering an adjunctive growth guided procedure like epiphyseodesis of the upper lateral tibial epiphysis for high grade group (grade V and VI) to be carried on together with the correction of the deformity. It must be taken in mind to achieving over-lengthening to address shortening which is the expected side effect of epiphyseodesis.

## Competing interests

The authors declare that they have no competing interests.

## Authors’ contributions

KAA collected the data, made all the measurements, analyzed the result, and drafted the manuscript. The author read and approved the final manuscript.
